# Detection of Multiple Microorganisms in Ruminant Ticks in Senegal Using High‐Throughput Microfluidic Real‐Time PCR

**DOI:** 10.1155/tbed/6292857

**Published:** 2026-02-20

**Authors:** Aliou Khoule, Clemence Galon, Déthié Ngom, Baye Bado Ndoye, Ousseynou Sene, Ibrahima Dia, Gamou Fall, Mawlouth Diallo, Sara Moutailler, Diawo Diallo

**Affiliations:** ^1^ Pôle de Zoologie Médicale, Institut Pasteur de Dakar, 36 Avenue Pasteur, Dakar, BP220, Senegal, pasteur.sn; ^2^ ANSES, INRAE, Ecole Nationale Vétérinaire d’Alfort, UMR BIPAR, Laboratoire de Santé Animale, Maisons-Alfort, F-94700, France, vet-alfort.fr; ^3^ Pôle de Virologie, Institut Pasteur de Dakar, 36 Avenue Pasteur, Dakar, BP220, Senegal, pasteur.sn

**Keywords:** co-infection prevalence, *Ehrlichia canis*, *Francisella*-like endosymbionts, microfluidic PCR, *Rickettsia aeschlimannii*, Senegal, small ruminants, tick-borne pathogens

## Abstract

Ticks are major vectors of numerous pathogens affecting both livestock and humans. In Senegal, data on the diversity of tick‐borne pathogens (TBPs) in ruminant‐associated ticks remain limited. In total, 1703 ticks were collected from goats, sheep, and cattle across three ecological zones of Senegal (Sudanian, Sahelian, and Sudano‐Sahelian). Tick species were identified morphologically, and 300 individuals were screened for 36 microorganisms using a high‐throughput microfluidic real‐time PCR system. DNA was successfully extracted and amplified from 289 ticks. The most abundant species were *Rhipicephalus evertsi evertsi* (32.3%), *Hyalomma truncatum* (19.6%), *R. guilhoni* (15.6%), *H. rufipes* (11.6%), and *Amblyomma variegatum* (11.0%). Among the screened ticks, 226 (78.9%) were positive for at least one microorganism. True pathogens of veterinary and/or zoonotic importance included *Anaplasma ovis* (30.8%), *Coxiella* spp. (23.9%), *Rickettsia aeschlimannii* (13.1%), *Theileria* spp. (11.1%), and *Ehrlichia canis* (4.8%), with sporadic detections of *Anaplasma marginale*, *A. bovis*, and *Babesia* spp. (0.3% each). In addition, non‐pathogenic *Francisella*‐like endosymbionts (FLEs) were detected at high prevalence (37.4%) across all ecological zones. The presence of TBPs and/or endosymbionts was significantly associated only with the tick’s host in the multivariable logistic regression model. Ticks collected from goats (OR = 7.82; *p* = 0.024) and sheep (OR = 7.70; *p* = 0.015) were significantly more likely to be infected than those collected from cattle (reference group). A total of 96 cases of microorganism co‐occurrence were recorded across different tick species. Co‐infections were more frequent in ticks collected from the Sudano‐Sahelian zone (48.2%) and in those from sheep (32.0%). None of the detected microorganism species showed a significant associated with tick sex. This study represents the first large‐scale molecular survey of TBPs in ruminant‐associated ticks in Senegal, revealing both a high diversity of pathogens and a widespread presence of tick endosymbionts. While endosymbionts, such as FLEs, are not known to be pathogenic, their abundance may influence tick physiology and vector competence. The detection of zoonotic pathogens, such as *E. canis* and *R. aeschlimannii*, underscores the need to strengthen tick surveillance and investigate their potential public health implications.

## 1. Introduction

Ticks are hematophagous arthropods and vectors of several pathogens of medical and veterinary importance. They are known to transmit a wide range of tick‐borne pathogens (TBPs), including bacteria (*Borrelia* and *Rickettsia*), protozoa (*Babesia* and *Theileria*), and viruses (Crimean‐Congo hemorrhagic fever virus; CCHFV) [1–3]. Their vector capacity is enhanced by several biological and ecological factors, including prolonged blood feeding, a wide range of vertebrate hosts, and the ability to acquire and transmit multiple pathogens simultaneously. Tick‐borne co‐infections can exacerbate disease severity or lead to atypical clinical symptoms, thereby complicating diagnosis and treatment [4].

Tick‐borne diseases (TBDs) are of global concern and represent a major burden to both public and animal health. Over 80% of the global cattle population is estimated to be at risk of TBDs, with sub‐Saharan Africa particularly vulnerable due to the negative impacts on livestock productivity and rural livelihoods [5]. In West Africa, the epidemiology of TBDs remains poorly documented. Limited access to diagnostic tools, low awareness among healthcare professionals and farmers, and a general scarcity of data contribute to an underestimation of TBP prevalence in both humans and animals [6].

Environmental changes, particularly the severe droughts in the late 20^th^ century that caused a 20% decline in annual rainfall, have disrupted traditional pastoral systems in the Sahel [7, 8]. These disruptions led to the widespread displacement of herders toward southern regions and the emergence of agro‐pastoralism systems, which increased the frequency of contact between animals with unknown immunological and infection statuses [9]. Such dynamics have facilitated the emergence and re‐emergence of TBDs and contributed to the dissemination of TBPs in this region [10]. For instance, tick‐borne relapsing fever caused by *Borrelia crocidurae* is the second most common cause of febrile illness after malaria in rural West African populations [11]. Moreover, arthropod‐borne borreliosis and rickettsiosis were detected in 16.3% of acute febrile cases reported by rural health centers and accounted for 11% of medical consultations in certain Senegalese villages [12].

Although several studies on ticks and TBPs have been conducted in Senegal [13, 14], most have focused on a limited spectrum of known pathogens. Earlier investigations predominantly relied on microscopic examination, which has low sensitivity and specificity [15], or on targeted PCR assays conducted in geographically restricted settings [14, 16]. As a result, the overall diversity and distribution of TBPs in the country remain largely unknown. Furthermore, among the 33 tick species associated with domestic animals in Senegal, many have never been screened for specific pathogens [17].

Recent advances in high‐throughput molecular diagnostic techniques offer promising opportunities for comprehensive surveillance of TBPs. Microfluidic real‐time PCR platforms allow the simultaneous detection of a broad array of pathogens in hundreds of samples per run, with high sensitivity and specificity [18–20]. These technologies are particularly well‐suited for integrated pathogen surveillance in settings characterized by environmental change and complex host–vector–pathogen interactions.

In this study, we present the first application of a microfluidic real‐time PCR system for large‐scale screening of bacteria and protozoa in field‐collected ticks in Senegal. The investigation was conducted across Sahelian, Sudano‐Sahelian, and Sudanian bioclimatic zones. The objectives were to detect both expected and novel TBPs, characterize their spatial distribution and contribute to a better understanding of the epidemiology of TBDs in Senegal.

## 2. Materials and Methods

### 2.1. Study Areas

Ticks were collected across three distinct bioclimatic zones of Senegal: the Sahelian, Sudano‐Sahelian, and Sudanian zones [21]. The Sahelian zone, in the northern part of the country, is the driest region, with a prolonged dry season lasting 8–9 months. Overgrazing and limited vegetation led to severe pasture degradation, promoting widespread transhumance in search of better grazing conditions. The Sudano‐Sahelian zone serves as a transition between the arid north and the more humid southern regions. It lies between the 600 and 1000 mm isohyets and supports both agricultural and pastoral activities. The Sudanian zone is the wettest and most ecologically diverse area of the country, receiving more than 1300 mm of annual rainfall. It has a rainy season of approximately 6 months and is characterized by dense vegetation that supports both crop farming and extensive livestock rearing.

### 2.2. Tick Collection and Treatment

Ticks were randomly collected in 2023 monthly (January, February, May, July, August, September, October, and November) from domestic animals (cattle, sheep, and goats). Animals were examined with the consent and cooperation of their owners, and ticks manually removed using sterile forceps. Following collection, ticks were morphologically identified to species level under a binocular microscope using standard taxonomic keys and morphometric tables [22, 23]. Specimens were transported in liquid nitrogen from the field and stored at −80°C at the Zoology Medical Laboratory of the Pasteur Institute of Dakar until further processing.

For molecular microfluidic assay analysis, a subset of 300 ticks previously preserved in 70% ethanol was randomly selected based on bioclimatic zone, host species and tick species. These samples were sent to ANSES (French Agency for Food, Environmental and Occupational Health & Safety in France) for screening.

### 2.3. DNA Extraction

Ticks were retrieved from ethanol, washed three times in sterile distilled water, and dried on sterile filter paper, to remove ethanol which can inhibit PCR reactions. They were then stored at −20°C. For tissue disruption, each tick was dissected into 2–4 parts using sterile scalpel and Petri dish. Dissection tools were decontaminated between samples to avoid cross‐contamination. Then, individual tick fragments were placed in tubes containing six stainless steel beads and 180 μL of lysis buffer.

The samples were homogenized twice for 20 s at 5500 rpm using the Precellys tissue homogenizer (Bertin, France), followed by centrifugation at 11,000 × *g* for 15 min. Finally, the lysate was then transferred to a 2 mL tube, and 25 μL of Proteinase K was added. DNA was then extracted for each sample using the NucleoSpin Tissue kit (Macherey‐Nagel, Germany), following the manufacturer’s instructions.

### 2.4. DNA Pre‐Amplification and High‐Throughput Microfluidic Real‐Time PCR

All samples were initially subjected to pre‐amplification using PreAmp Master Mix to enhance detection sensitivity [24]. A pooled primer mix targeting 36 pathogens (Supporting Information 1: Table S1) was used, with each primer at a final concentration of 0.2 μM. The pre‐amplification reaction was performed in a total volume of 5 μL, consisting of 1.25 μL DNA, 1.25 μL primers, 1 μL master mix, and 1.5 μL of water ran 14 cycles (95°C × 15 s, 60°C × 4 min) after an initial denaturation at 95°C × 2 min. Products were diluted 1:10 and stored at –20°C.

Pathogen detection then used a high‐throughput BioMark 48.48 Dynamic Array for real‐time PCR (2304 simultaneous assays), with PerfeCTa qPCR ToughMix Low ROX and FAM/BHQ1 probes under standard cycling (50°C × 2 min; 95°C × 10 min; 40 × [95°C × 15 s, 60°C × 1 min]). Each chip included water blanks and an *E. coli* inhibition control. Primers and probes are detailed in Supporting Information 2: Table S2.

### 2.5. Standard/Nested PCR and Sequencing

Samples were considered positive for a given microorganism if the *C*_p_ value was <30, and if they were positive for both the target pathogen species and its corresponding genus, while remaining negative for all other species within the same genus. Samples yielding ambiguous or unexpected results were subjected to confirmation using nested or conventional PCR with primers targeting genes or genomic region not included in the BioMark system (primers sequences are listed in Supporting Information 3: Table S3). PCR amplicons were sequenced by Eurofins Genomics (Germany). The resulting sequences were assembled and edited using BioEdit software (Ibis Biosciences, Carlsbad, CA, USA). Sequence identities were determined by comparison with publicly available sequences using BLASTN algorithm against the NCBI GenBank nucleotide database (https://www.ncbi.nlm.nih.gov/, accessed on December 15, 2024).

### 2.6. Statistical Analysis

A multivariable logistic regression analysis was performed to identify factors associated with the presence of TBPs or endosymbionts. The dependent variable was the presence/absence of TBPs/endosymbionts (binary outcome), while explanatory variables included geographical zone, host species, tick species and sex. Prior to model fitting, a data filtering step was applied to ensure robust estimations. Tick species with a total count of fewer than five individuals (*n* < 5) or showing no variability in infection status (i.e., 100% positive or 100% negative) were excluded, as such cases can lead to infinite coefficient estimates and poor model convergence. The model was fitted using a binomial distribution with the glm () function in R software. Model selection and evaluation were based on the following criteria: the Akaike information criterion (AIC) to assess model parsimony, the area under the ROC curve (AUC) to evaluate predictive performance, and the variance inflation factor (VIF) to assess multicollinearity among predictors.

We assessed the overall prevalence of infection, as well as the prevalence of each pathogen or endosymbiont by tick species, sex, host species, and geographic origin, with corresponding 95% confidence intervals (95% CIs) based on the binomial distribution. Association between pathogen presence and categorical variables were evaluated using the Chi‐square test or Fisher’s exact test, as appropriate. All statistical tests were performed using R software. Differences were considered statistically significant when *p*  < 0.05.

## 3. Results

### 3.1. Tick Abundance and Species Diversity

A total of 1703 ticks were collected from domestic animals across three ecological zones of Senegal. Among these, 300 ticks were selected for pathogen screening (Table 1): 205 from the Sahelian zone (seven species), 67 from the Sudanian zone (eight species), and 28 from the Sudano‐Sahelian zone (10 species). The most abundant species were *Rhipicephalus evertsi evertsi* (32.2%), *Hyalomma truncatum* (19.6%), *R. guilhoni* (15.6%), *H. rufipes* (11.6%), *Amblyomma variegatum* (11.0%), *H. impeltatum* (3.0%), *R. sulcatus* (1.7%), *H. impressum* (1.3%), *R*. (*Boophilus*) *geigyi* (1.3%), *R*. (*B*.) *decoloratus* (1.0%), *R. lunulatus* (0.7%), *R. senegalensis*, and *R. sanguineus* (0.3%).

**Table 1:** Distribution of the 300 ticks analyzed by microfluidic real‐time PCR according to host species and ecological zone.

Tick species	Sahelian			Sudanian			Sudano‐Sahelian			Total (%)
	Goats	Sheep	Cattle	Goats	Sheep	Cattle	Goats	Sheep	Cattle	
*A. variegatum*	0	0	0	0	0	27	0	0	6	33 (11.0)
*H. impeltatum*	0	6	2	0	0	0	0	0	1	9 (3.0)
*H. impressum*	0	0	0	0	0	3	0	0	1	4 (1.3)
*H. rufipes*	0	3	2	0	0	25	1	0	4	35 (11.6)
*H. truncatum*	7	38	7	0	0	4	0	0	3	59 (19.6)
*R. evertsi*	42	50	0	0	0	0	0	3	2	97 (32.3)
*R. guilhoni*	23	23	0	0	0	0	0	0	1	47 (15.6)
*R. lunulatus*	0	0	0	0	0	2	0	0	0	2 (0.7)
*R. sanguineus*	0	0	0	0	0	1	0	0	0	1 (0.3)
*R. senegalensis*	0	0	0	0	0	1	0	0	0	1 (0.3)
*R. sulcatus*	0	0	0	0	0	0	0	3	2	5 (1.7)
*R*. (*B*.) *geigyi*	0	0	0	0	0	4	0	0	0	4 (1.3)
*R*. (*B*.) *decoloratus*	0	2	0	0	0	0	0	0	1	3 (1.0)
Total	72	122	11	0	0	67	1	6	21	300 (100)

Abbreviations: *A*., *Amblyomma*; *H*., *Hyalomma*; *R*., *Rhipicephalus*; *R*. (*B*.), *Rhipicephalus* (*Boophilus*).

### 3.2. Prevalence and Analysis of Potential Factors Associated With the Presence of TBPs/Endosymbionts

#### 3.2.1. Univariable Analysis

Univariable logistic regression identified several factors potentially associated with the presence of TBPs or endosymbionts. Ticks collected from sheep (OR = 2.1, 95% CI: 1.1–4.0, *p* = 0.032) and those belonging to *H. rufipes* (OR = 51.3, 95% CI: 2.9–908.0, *p* < 0.001) or *H. truncatum* (OR = 4.5, 95% CI: 1.7–12.2, *p* = 0.003) showed significantly higher crude odds of TBP/endosymbionts detection compared to their respective reference groups (cattle and *A. variegatum*). Ticks collected from the Sudanian zone were less likely to be positive than those from the Sahelian zone (OR = 0.5, 95% CI: 0.2–0.9, *p* = 0.013). No significant difference was observed according to tick sex (*p* = 0.391).

#### 3.2.2. Multivariable Logistic Regression Model

After excluding tick species with low sample size or no infection variability (100% positive or negative), a total of 236 ticks representing five species were included in the multivariable analysis. The final logistic regression model, including zones, hosts, sex, and species showed a good model fit (AIC = 247.4) and a moderate discriminative ability with an AUC = 0.7089. All predictors presented low multicollinearity, with VIF values below 2 (mean VIF = 1.4). Among the tested variables, the host type was identified as a significant predictor of TBP presence/symbiont. Ticks collected from goats (OR = 7.82; *p* = 0.024) and sheep (OR = 7.70; *p* = 0.015) were significantly more likely to be infected compared to ticks collected from cattle (reference category). No significant association was found for zone, sex, or tick species (*p*  > 0.05), although a slight trend was observed for ticks from the sudano‐sahelian zone and for female ticks (Table 2).

**Table 2:** Results of univariable and multivariable logistic regression analyses assessing factors associated with the presence of tick‐borne pathogens (TBPs) and endosymbionts in ruminant ticks collected in Senegal, 2023.

Factors		Total tested (*N*)	Positif (% [95% CI])	Crude ORs (95% CI)	*p*‐Value	Ajusted ORs (95% CI)	*p*‐Value
Zones	Sahelian	198	162 (81.8%, [75.7–86.9])	Ref	—	—	—
	Sudanian	65	43 (66.2%, [53.4–77.4])	0.5 (0.2–0.9)	0.013^a^	1.2 (0.1–9.5)	0.893
	Sudano‐sahelian	26	23 (88.5%, [69.8–97.6])	1.5 (0.5–4.9)	0.648	3.2 (0.5–25.5)	0.234
Hosts	Cattle	96	68 (70.8%, [60.7–79.7])	Ref	—	Ref	—
	Goats	72	58 (80.6%, [69.5–88.9])	1.6 (0.8–3.3)	0.178	7.8 (1.3–49.2)	0.024^a^
	Sheep	121	102 (84.3%, [76.6–90.3])	2.1 (1.1–4.0)	0.032^a^	7.7 (1.5–42.7)	0.015^a^
Species	*A. variegatum*	33	18 (54.5%, [36.4–71.9])	Ref	—	Ref	—
	*H. impeltatum*	9	8 (88.9%, [51.8–99.7])	4.2 (0.7–27.2)	0.25	2.9 (0.2–83.9)	0.449
	*H. impressum*	4	4 (100.0%, [39.8–100.0])	6.7 (0.3–134.0)	0.14	—	—
	*H. rufipes*	34	34 (100.0%, [89.7–100.0])	51.3 (2.9–908.0)	<0.001^a^	—	—
	*H. truncatum*	59	51 (86.4%, [75.0–94.0])	4.5 (1.7–12.2)	0.003^a^	1.7 (0.3–9.5)	0.554
	*R*. (*B*). *geigyi*	4	1 (25.0%, [0.6–80.6])	0.3 (0.0–2.4)	0.29	—	—
	*R*. (*B*). *decoloratus*	3	3 (100.0%, [29.2–100.0])	5.2 (0.3–109.0)	0.14	—	—
	*R. evertsi*	90	67 (74.4%, [64.2–83.1])	2.1 (0.9–4.9)	0.11	0.4 (0.1–2.4)	0.301
	*R. guilhoni*	45	38 (84.4%, [70.5–93.5])	3.8 (1.4–10.8)	0.02	0.7 (0.1–5.4)	0.735
	*R. lunulatus*	2	0 (0.0%, [0.0–84.2])	0.2 (0.0–3.3)	0.19	—	—
	*R. sanguineus*	1	0 (0.0%, [0.0–97.5])	0.3 (0.0–6.5)	0.19	—	—
	*R. senegalensis*	1	0 (0.0%, [0.0–97.5])	0.3 (0.0–6.5)	0.19	—	—
	*R. sulcatus*	4	4 (100.0%, [39.8–100.0])	6.9 (0.33–134.3)	0.14	—	—
Sex	F	106	87 (82.0%, [73.4–88.8])	Ref	—	Ref	—
	M	183	142 (77.6, [70.8–83.4])	0.8 (0.4–1.4)	0.391	0.5 (0.3–1.1)	0.099

*Note*: Crude ORs (95% CI) were obtained from univariable logistic regression models. Adjusted ORs (95% CI) were derived from the final multivariable logistic regression model, including the variables “zones,” “hosts,” “sex,” and “tick species.” Tick species with fewer than five individuals or with no variability in infection status (i.e., 100% positive or 100% negative) were excluded from the model.

Abbreviations: *A*., *Amblyomma*; *H*., *Hyalomma*; R., *Rhipicephalus*; *R*. (*B*)., *Rhipicephalus* (*Boophilus*); Ref, reference.

^a^Significant *p*‐value <0.05.

### 3.3. Prevalence and diversity of tick‐borne Pathogens/Endosymbionts

A total of nine microorganisms were detected in the 289 ticks (Table 3). *Francisella*‐like endosymbionts (FLEs) were the most frequently detected (37.4%; 108/289), found in eight tick species from all hosts and ecological zones. FLE prevalence significantly varied by tick species (from 0% in *Rhipicephalus* spp. to 100% in *H. impressum*), host (goats: 19.4%, sheep: 38.8%, and cattle: 49.0%) (*p* < 0.001; Fisher’s exact test), and slightly by ecological zones (Sahelian zone: 33.3%, Sudano‐Sahelian: 34.6% Sudanian zone: 50.8%) (*p* = 0.04; Chi‐square test).

**Table 3:** Prevalence of infection with microorganisms (bacteria and parasites) and 95% binomial exact confidence intervals of ruminant ticks in Senegal, 2023.

Factors		Number tested	Number of positive samples (%, binomial 95% confidence intervals)									
			*Babesia* spp.	*Anaplasma bovis*	*Anaplasma marginale*	*Anaplasma ovis*	*Coxiella*. spp.	*Ehrlichia canis*	FLE	*Rickettsia aeschlimannii*	*Theileria* spp.	Overall
Species	*A. variegatum*	33	1 (3.0%, [0.1–15.8])	0	0	0	6 (18.2%, [7.0–35.5])	0	5 (15.2%, [5.1–31.9])	5 (15.2%, [5.1–31.9])	5 (15.2%, [5.1–31.9])	18 (54.5%, [36.4–71.9])
	*H. impeltatum*	9	0	0	0	1 (11.1%, [0.3–48.2])	5 (55.6%, [21.2–86.3])	0	8 (88.9%, [51.8–99.7])	0	0	8 (88.9%, [51.8–99.7])
	*H. impressum*	4	0	0	0	0	1 (25.0%, [0.6–80.6])	0	4 (100.0%, [39.8–100.0])	1 (25.0%, [0.6–80.6])	0	4 (100.0%, [39.8–100.0])
	*H. rufipes*	34	0	0	0	0	5 (14.7%, [5.0–31.1])	0	32 (94.1%, [80.3–99.3])	20 (58.8%, [40.7–75.4])	3 (8.8%, [1.9–23.7])	34 (100.0%, [89.7–100.0])
	*H. truncatum*	59	0	0	0	13 (22.0%, [12.3–34.7])	17 (28.8%, [17.8–42.1])	0	44 (74.6%, [61.6–85.0])	8 (13.6%, [6.0–25.0])	3 (5.1%, [1.1–14.1])	51 (86.4%, [75.0–94.0])
	*R*. (*B*.) *geigyi*	4	0	0	1 (25.0%, [0.6–80.6])	0	0	0	0	0	1 (25.0%, [0.6–80.6])	1 (25.0%, [0.6–80.6])
	*R*. (*B*.) *decoloratus*	3	0	0	0	0	2 (66.7%, [9.4–99.2])	0	1 (33.3%, [0.8–90.6])	1 (33.3%, [0.8–90.6])	0	3 (100.0%, [29.2–100.0])
	*R. evertsi*	90	0	1 (1.1%, [0.0–6.0])	0	40 (44.4%, [34.0–55.3])	19 (21.1%, [13.2–31.0])	12 (13.3%, [7.1–22.1])	13 (14.4%, [7.9–23.4])	3 (3.3%, [0.7–9.4])	16 (17.8%, [10.5–27.3])	67 (74.4%, [64.2–83.1])
	*R. guilhoni*	45	0	0	0	34 (75.6%, [60.5–87.1])	10 (22.2%, [11.2–37.1])	2 (4.4%, [0.5–15.1])	1 (2.2%, [0.1–11.8])	0	3 (6.7%, [1.4–18.3])	38 (84.4%, [70.5–93.5])
	*R. lunulatus*	2	0	0	0	0	0	0	0	0	0	0 (0.0%, [0.0–84.2])
	*R. sanguineus*	1	0	0	0	0	0	0	0	0	0	0 (0.0%, [0.0–97.5])
	*R. senegalensis*	1	0	0	0	0	0	0	0	0	0	0 (0.0%, [0.0–97.5])
	*R. sulcatus*	4	0	0	0	1 (25.0%, [0.6–80.6])	4 (100.0%, [39.8–100.0])	0	0	0	1 (25.0%, [0.6–80.6])	4 (100.0%, [39.8–100.0])
Zones	Sahelian	*198*	162 (81.8%, [75.7–86.9])	0	1 (0.5%, [0.0–2.8])	0	87 (43.9%, [36.9–51.2])	46 (23.2%, [17.5–29.7])	13 (6.6%, [3.5–11.0])	66 (33.3%, [26.8–40.4])	9 (4.5%, [2.1–8.5])	23 (11.6%, [7.5–16.9])
	Sudanian	*65*	43 (66.2%, [53.4–77.4])	1 (1.5%, [0.0–8.3])	0	1 (1.5%, [0.0–8.3])	0	2 (3.1%, [0.4–10.7])	0	33 (50.8%, [38.1–63.4])	20 (30.8%, [19.9–43.4])	8 (12.3%, [5.5–22.8])
	Sudano‐sahelian	*26*	23 (88.5%, [69.8–97.6])	0	0	0	2 (7.7%, [0.9–25.1])	21 (80.8%, [60.6–93.4])	1 (3.8%, [0.1–19.6])	9 (34.6%, [17.2–55.7])	9 (34.6%, [17.2–55.7])	1 (3.8%, [0.1–19.6])
Hosts	Cattle	*96*	68 (70.8%, [60.7–79.7])	1 (1.0%, [0.0–5.7])	0	1 (1.0%, [0.0–5.7])	1 (1.0%, [0.0–5.7])	20 (20.8%, [13.2–30.3])	1 (1.0%, [0.0–5.7])	47 (49.0%, [38.6–59.4])	28 (29.2%, [20.3–39.3])	8 (8.3%, [3.7–15.8])
	Goats	*72*	58 (80.6%, [69.5–88.9])	0	1 (1.4%, [0.0–7.5])	0	39 (54.2%, [42.0–66.0])	13 (18.1%, [10.0–28.9])	7 (9.7%, [4.0–19.0])	14 (19.4%, [11.1–30.5])	3 (4.2%, [0.9–11.7])	7 (9.7%, [4.0–19.0])
	Sheep	*121*	102 (84.3%, [76.6–90.3])	0	0	0	49 (40.5%, [31.7–49.8])	36 (29.8%, [21.8–38.7])	6 (5.0%, [1.8–10.5])	47 (38.8%, [30.1–48.1])	7 (5.8%, [2.4–11.6])	17 (14.0%, [8.4–21.5])
	Overall prevalence	289	1 (0.3%, [0.0–1.9])	1 (0.3%, [0.0–1.9])	1 (0.3%, [0.0–1.9])	89 (30.8%, [25.5–36.5])	69 (23.9%, [19.1–29.2])	14 (4.8%, [2.7–8.0])	108 (37.4%, [31.8–43.2])	38 (13.1%, [9.5–17.6])	32 (11.1%, [7.7–15.3])	228 (78.9%, [73.7–83.5])

Abbreviations: *A*., *Amblyomma*; FLE, *Francisella*‐like endosymbiont; *H*., *Hyalomma*; *R*., *Rhipicephalus*; *R*. (*B*.), *Rhipicephalus* (*Boophilus*).

*Anaplasma ovis* was the second most prevalent pathogen (30.8%; 89/289), predominantly detected in *R. guilhoni* (75.6%; 34/45) and *R. evertsi* (44.4%; 40/90). The highest prevalence was observed in ticks from goats (54.2%) and in the Sahelian zone (43.9%), with significant differences by tick species, host, and ecological zone (*p* < 0.001; Fisher’s exact test).

*Coxiella* spp. infected 23.9% of ticks (69/289). It was detected in nine species, including: *R. sulcatus* (100%; 4/4), *R*. (*B*) *decoloratus* (66.7%; 2/3), and *H. impeltatum* (55.6%; 5/9). Prevalence differed significantly by tick species (*p* < 0.001). The pathogen was more prevalent in the Sudano‐Sahelian zone (80.8%; 21/26) compared to the Sahelian (23.2%; 46/198) and Sudanian zone (3.1%; 2/65) (*p* < 0.001; Fisher test). However, no significant association was found between prevalence and host species (*p* = 0.1; Chi‐square test).

*Rickettsia aeschlimannii* (13.1%, 38/289) was detected in six tick species. Prevalence significantly varied by tick species, host origin, and ecological zone (*p* < 0.001; Fisher’s exact test), with the highest rates in *H. rufipes* (58.8%; 20/34), in ticks from cattle (29.2%), and in ticks from the Sudano‐Sahelian (34.6%) and Sudanian (30.8%) zones.

*Theileria ovis* was detected in 11.1% (32/289) of ticks from seven species. However, no statistically significant differences in prevalence were observed by tick species, host, or ecological zone (*p* > 0.05; Fisher’s exact test).

*Ehrlichia canis* was exclusively detected in *R. evertsi* (13.3%; 12/90) and *R. guilhoni* (4.4%; 2/45), mainly from goats (9.7%) in the Sahelian zone.

*Anaplasma marginale*, *A. bovis*, and *Babesia* spp. were each detected at low prevalence (0.3%). *A. marginale* and *Babesia* spp. were found in *R*. (*B*.) *geigyi*, *R. evertsi*, and *A. variegatum* collected from cattle in the Sudanian zone, while *A. bovis* was detected in a tick collected from a goat in the Sahelian zone.

### 3.4. Infection Types

**Figure 1:**
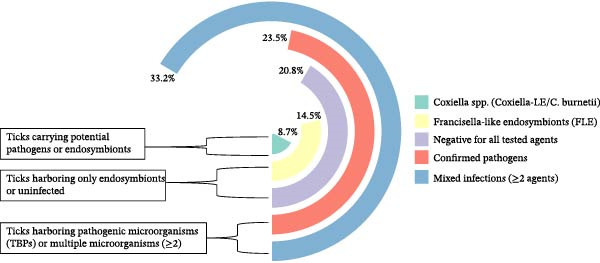
Distribution of infection types among ruminant ticks in 2023, Senegal.

To better distinguish between pathogenic and non‐pathogenic microorganisms, the detected agents were classified into five categories (Figure 1): confirmed TBPs (*Anaplasma*, *Babesia*, *Ehrlichia*, *Rickettsia*, and *Theileria*), FLEs considered non‐pathogenic, *Coxiella* spp. (*Coxiella*‐LE */C. burnetii*), which may represent either *Coxiella burnetii* (pathogenic) or *Coxiella*‐like endosymbionts (CLE; non‐pathogenic), mixed infections (≥2 agents), and negative samples. Overall, 79.2% of ticks carried at least one microorganism, whereas 20.8% tested negative for all agents. Co‐infections were the most frequent (33.2%), followed by confirmed pathogens (23.5%), FLE (14.5%), and *Coxiella* spp. (8.7%). These findings emphasize the coexistence of both symbiotic and pathogenic microorganisms within tick populations.

### 3.5. Prevalence of Co‐Infections

**Figure 2:**
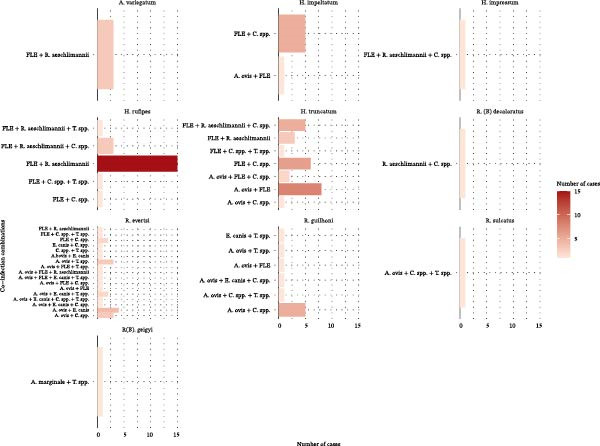
Co‐infection patterns of tick‐borne pathogens/endosymbionts detected in various tick species.

Among 289 positive ticks, 96 (33.2%) were co‐infected by multiple microorganisms (Figure 2). The highest co‐infection rates were observed in *Hyalomma* species, particularly in *H. impeltatum* (6/9; 66.7%), *H. rufipes* (21/34; 61.8%), and *H. truncatum* (26/59; 44.1%).

### 3.6. Co‐Infection Combinations

The most frequent co‐infection identified was *R. aeschlimannii* + FLE, accounting for 19.9% (19/96) of all co‐infection cases (Figure 2). This combination was detected in *A. variegatum* (*n* = 3), *H. rufipes* (*n* = 15), and *R. evertsi* (*n* = 1). The combination of FLE + *Coxiella* spp. (14.6%) was found in *H. impeltatum* (*n* = 5), *H. rufipes* (*n* = 1), *H. truncatum* (*n* = 6), and *R. evertsi* (*n* = 2). Triple infections involving FLE, *Coxiella* spp., and *R. aeschlimannii* were detected in 8.4% of cases, occurring in *H. impressum*, *H. rufipes*, and *H. truncatum*. Additionally, two quadruple co‐infections were recorded in *R. evertsi*, involving *A. ovis*, *E. canis*, *Theileria* spp., and either *Coxiella* spp. or FLE. *Coxiella* spp. was the microorganism most frequently involved in co‐infections, being present in 45.3% of all cases.

### 3.7. Prevalence of Co‐Infection by Ecozone and Host Species

The prevalence of co‐infection was evaluated across different ecological zones and host (Figure 3). The Sudano‐Sahelian zone showed the highest prevalence (48.2%), followed by the Sahelian zone (32.8%) and the Sudanian zone (29.2%). However, there was no statistically significant association between co‐infection cases and the ecological origin of ticks (*p* = 0.295, Chi‐square test). Regarding host species, the highest co‐infection prevalence was observed in ticks collected from sheep (38.0%), followed by those from cattle (31.2%), and finally goats (27.8%). Nonetheless, the difference was not statistically significant (*p* = 0.295, Chi‐square test)

Figure 3Distribution of co‐infection prevalence in ticks, 2023, Senegal, based on (a) ecological zones (Sahelian, Sudano‐Sahelian, and Sudanian) and (b) host species.

**Figure:**
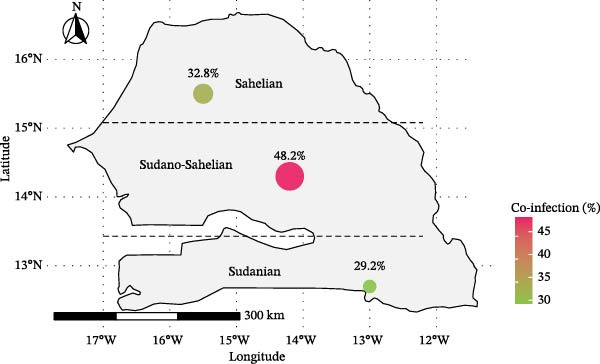
(a)

**Figure:**
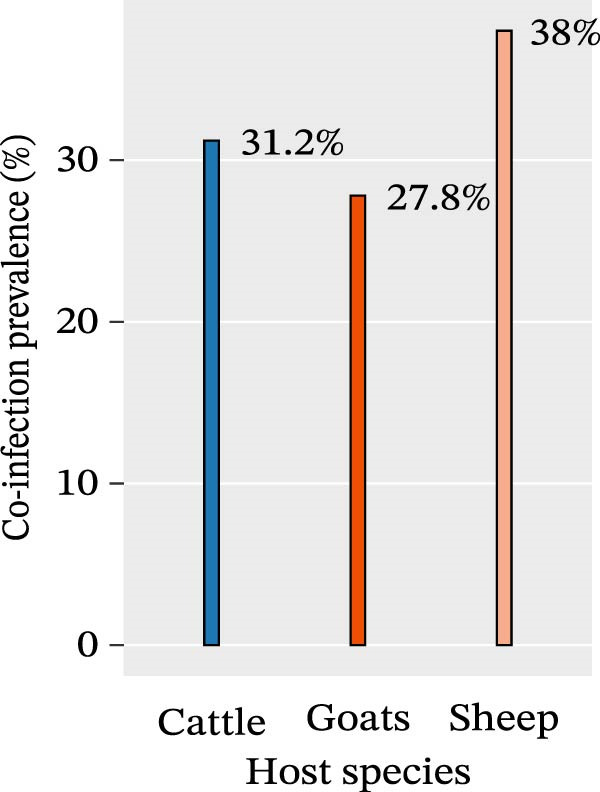
(b)

### 3.8. Prevalence of TBPs/Endosymbionts in Male and Female Ticks

**Figure 4:**
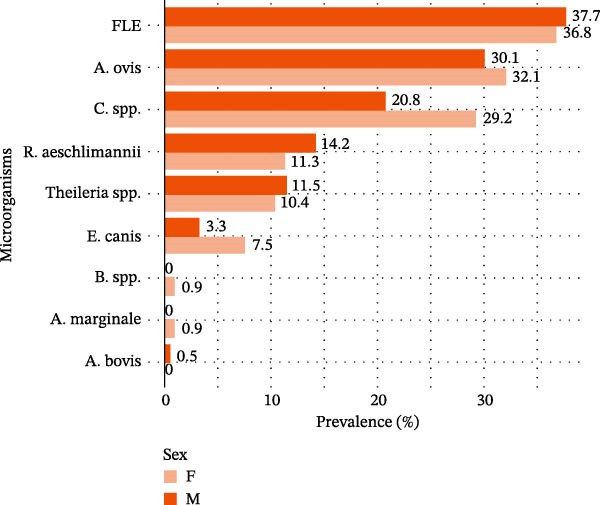
Prevalence of microorganism species according to tick sex, 2023, Senegal.

Globally prevalence of infection was slightly higher in female ticks (82.0%, 87/106) compared to males (77.6%, 142/183). The results presented in Figure 4 showed that *A. marginale* and *Babesia* spp. were detected exclusively in female ticks, while *A. ovis*, *Coxiella* spp., and *E. canis* were also more frequently found in females. Conversely, FLE, *R. aeschlimannii*, and *Theileria* spp. were slightly more prevalent in male ticks. Despite these differences, no statistically significant association was observed between tick sex and overall infection status (*p*  > 0.1).

## 4. Discussion

TBDs represent a major concern for public health and veterinary health in tropical regions. In Africa, they include infections caused by bacteria (tick‐borne spotted fever, relapsing fever, anaplasmosis, ehrlichiosis, bartonellosis, and Q fever), parasites (theileriosis and babesiosis), viruses (Crimean‐Congo hemorrhagic fever and African swine fever) [25]. In West Africa, the detection of pathogens in ticks has traditionally relied on classical diagnostic methods, such as blood smears, standard PCR, or real‐time PCR, which limit the capacity to simultaneously identify co‐infections despite their known epidemiological relevance [26]. Recent advances in diagnostic platforms, such as microfluidic high‐throughput PCR, allow for the detection of multiple pathogens in a single reaction [20, 27]. While the high‐throughput microfluidic real‐time PCR platform used in our study enabled sensitive, multiplex detection of several TBPs, it remains limited by its dependence on predefined primer–probe sets. Consequently, some microorganisms could only be identified at the genus level. In contrast, the genome‐resolved metagenomic approach applied by Du et al. [28] provides a broader and more detailed picture of tick microbiomes, enabling species and even strain‐level identification of both pathogens and endosymbionts without prior target selection. However, metagenomics requires more complex bioinformatic pipelines and higher sequencing costs, making microfluidic PCR a more accessible option for large‐scale epidemiological surveillance in resource‐limited settings such as West Africa.

Our study is the first in Senegal to apply this high‐throughput technology, enabling the multiplex detection of nine tick microorganisms (seven bacterial species and two protozoa) in individual ticks. This represents the greatest diversity of TBPs ever reported in the country to date. The overall infection rate of 78.9% is comparable to values reported from North African countries using similar technologies [29, 30], and exceeds previously reported rates in South America [27], highlighting the enhanced sensitivity of this approach.

Pathogen prevalence varied by ecological zone and host species, with the highest rates observed in the Sudano‐Sahelian region and in ticks collected from small ruminants. Our results revealed a higher prevalence of tick‐associated microorganisms compared with most previously reported studies from African countries [31]. The increased detection here likely reflects the improved sensitivity and multiplex capacity of the microfluidic PCR approach. The species‐specific differences observed in infection rates in this study (with *H. rufipes* exhibiting 100% infection, whereas *R. lunulatus*, *R. sanguineus*, and *R. senegalensis* were uninfected) are consistent with observations reported by Ouedraogo et al. [32]. Such interspecific variation likely reflects differences in vector competence, host preferences among tick species [33]. Similarly, Du et al. [28] reported that microbial community composition in ticks was primarily influenced by tick species and host identity rather than by geographical location. This supports our multivariable analysis, which identified the host as the only significant predictor of TBP/endosymbiont presence, further suggesting that host–tick interactions play a more decisive role than environmental factors in shaping pathogen occurrence, as also reported by Heylen et al. [34].

FLEs were detected at high prevalence (37%) across all three ecological zones. This represents the first confirmed report of widespread FLE presence in Senegalese ticks, consistent with findings from Algeria [30] and Tunisia [35] and exceeding prevalence reported in Egypt [36]. Several ecological mechanisms may explain the high abundance of FLE, which is commonly found as an endosymbiont in various tick species. As reported in previous studies, FLE is often associated with blood‐feeding ticks and may play a mutualistic role in their nutrition, particularly in species with restricted host ranges [37]. Although generally considered non‐pathogenic, the close phylogenetic relationship between pathogenic and mutualistic *Francisella* lineages, coupled with their potential influence on the tick microbiome and vector competence, warrants further investigation.

*A. ovis* was identified for the first time in ticks in West Africa, with a high prevalence (30.8%), particularly in *R. guilhoni* (75.6%) and *R. evertsi* (44.4%), suggesting a potential vector role for these species. *A. ovis* had previously been detected serologically in sheep in Senegal, with increasing seroprevalence over time [13, 15, 38], their detection in ticks supports ongoing transmission and the role of small ruminants as reservoirs. Similar results in East Africa, where *A. ovis* implicate *R. evertsi*, *Amblyomma* spp., and *H. marginatum* [39], as possible vectors reinforcing the importance of *R. evertsi* as a key vector [40]. In contrast, *A. marginale*, the agent of bovine anaplasmosis was detected in only one *R*. (*B*.) *geigyi* specimen, suggesting a low tick infection rate. This is consistent with results from Burkina Faso [32], although *A. marginale* is widespread West African cattle [6, 13, 15]. Its biological transmission involves at least 20 tick species primarily *Dermacentor* and *Rhipicephalus* [41].

Finally, *A. bovis* was detected for the first time in ticks in Senegal, and more broadly in West Africa, despite previous studies using broad‐range PCR assays. In contrast, *A. bovis* has been well documented in other regions, such as Tunisia, Kenya, and South Africa, where it has been identified in both livestock and ticks, with reported prevalence rates of up to 17.4% [42–44].

*E. canis*, the causative agent of canine monocytic ehrlichiosis, was detected in 4.8% of the ticks analyzed, specifically in *R. evertsi* (13.3%) and for the first time in *R. guilhoni* (4.4%). Unexpectedly, *R. sanguineus*, the main known vector in Africa, tested negative. This result is likely attributable to the collection of ticks that had fed on infected dogs during their immature stages. Previous investigations in Senegal have reported *E. canis* infection rates ranging from 18.8% to 53% in dogs [13], while ticks collected from dogs in neighboring countries, such as Nigeria (23.7%) and Cameroon (21%), have also shown notable infection rates [45, 46]. The relatively low prevalence observed in our samples could indicate regional variation in vector–host dynamics or differences in tick population structure.

*Coxiella* spp., a genus that includes *Coxiella burnetii*, the causative agent of Q fever, were detected in 8.7% of ticks across nine species. However, these detections must be interpreted at the genus level, as the sequencing quality obtained from PCR products targeting the IS1111/idc gene did not allow discrimination between pathogenic *C. burnetii* and CLE, even when positive signals were observed in microfluidic PCR. Consequently, all positive detections were conservatively classified as *Coxiella* spp. This distinction is epidemiologically important, given that the presence of *Coxiella*‐LE reflects the microbiome composition rather than a direct public health risk. High detection rates in *R. sulcatus* (100%), *R*. (*B*.) *decoloratus* (66.7%), and *H. impeltatum* (55.6%) suggest that *Coxiella* spp. may be widespread among ruminant‐associated ticks in Senegal. These findings support the hypothesis that *Coxiella*‐LEs are essential symbionts in many tick species, as reported in several studies [47]. Moreover, *Coxiella*‐LE are known to infect certain tick species at high rates [48] and to play essential roles as nutritional symbionts, contributing to host adaptation and reproductive fitness. Future studies employing more discriminative genetic markers (e.g., *com1*, *icd*, or 16S rRNA sequencing) or genome‐resolved metagenomics [28] are needed to confirm the presence of *C. burnetii* and to better characterize the diversity and evolutionary relationships of *Coxiella* symbionts in these populations.

*R. aeschlimannii*, a member of the spotted fever group (SFG) rickettsia and recognized agent of spotted fever, was detected in six tick species in this study, with an overall prevalence of 13.1%. This prevalence is more than double that reported by Sambou et al. [14]. The infection rate observed in *H. rufipes* (58.8%) is consistent with previous findings (44.8%–51.3%) in the Sudano‐Sahelian zone. This study broadens the known vector range of *R. aeschlimannii* in Senegal, which was previously limited to *H. rufipes* and *H. truncatum*. Other SFG Rickettsia species were expected to be detected, but their absence may be attributed to geographic variation or differences in the molecular detection targets. These findings also support the hypothesis that *R. aeschlimannii* may act as a symbiont in certain *Hyalomma* species [49].

In this study, *Theileria* spp. and *Babesia* spp. were detected in ticks for the first time in Senegal, with prevalences of 11.1% and 0.3%, respectively. The prevalence of *Theileria* spp. was notably higher than previous reports from Egypt, Ethiopia, and Kenya (2.1%; Olivieri et al. [50]), with infections more frequent in ticks from sheep (14.0%) and the Sudanian zone (12.3%), suggesting possible host or ecological factors. Several *Theileria* species have been reported in West Africa, including *T. annulata*, *T. mutans*, *T. ovis*, and *T. equi*, the latter three previously identified in Senegalese livestock. *Babesia bigemina* and *B. caballi* were previously reported in cattle and equines, respectively, but only in blood samples [13, 15]. Similar taxonomic limitations applied to *Babesia* spp. and *Theileria* spp., which were only identified to the genus level, underscoring the need for higher‐resolution molecular approaches to accurately assess the epidemiological relevance of these protozoan infections.

This study reports, for the first time in Senegal, a high prevalence of co‐infections with TBPs/endosymbionts, detected in 33.2% of ticks. This rate exceeds those reported in Côte d’Ivoire (4%), Nigeria (11.8%), and Cuba (9.3%) [27, 45]. Interestingly, co‐infections involving FLE and *Coxiella* spp. were detected across four tick species, consistent with the observations of Binetruy et al. [48], who reported this association in three tick species. However, our results show that the prevalence of co‐infections involving FLE and *Coxiella* spp. was relatively frequent (14.7%) particularly in *Hyalomma* ticks, which contrasts with the exclusion pattern also described by Binetruy et al. [48], where *Coxiella*‐LE and FLE rarely co‐occurred within the same *Amblyomma* species. This discrepancy may reflect differences in host ecology, tick genus, or microbial interactions that allow symbiont coexistence within *Hyalomma* species, in contrast to previous studies suggesting competitive interactions between these symbionts [47, 50].

The most common co‐infection detected was *R. aeschlimannii* and FLE, accounting for 19.9% of all co‐infection cases, consistent with previous reports [51, 52]. Triple co‐infections involving FLE, *Coxiella* spp., and *R. aeschlimannii* (8.4%) were identified exclusively in *Hyalomma* ticks, which may acquire multiple pathogens due to their three‐host life cycle and feeding on diverse vertebrate hosts [4, 53]. This hypothesis partially aligns with the findings of Binetruy et al. [48], who suggested that *Rickettsia* may establish infections independently of these endosymbionts, indicating complex and potentially species‐specific microbial interactions within ticks.

No significant difference in TBP/endosymbiont prevalence was observed between male and female ticks, in line with previous studies [1, 54]. Although Du et al. [28] recorded tick sex in their pooled‐sample design, they did not report any sex‐specific variation in microbial composition. This finding supports the hypothesis that several TBPs may be vertically transmitted, facilitating their persistence within tick populations. However, our results contrast with those of Kratou et al. [35] and Benyedem et al. [37], who reported distinct microbiota patterns between male and female *H. dromedarii* ticks.

The highest diversity and frequency of infections were recorded in ticks from the Sahelian and Sudano‐Sahelian zones, where transhumance, high livestock density, and environmental conditions promote tick proliferation and pathogen maintenance. *Hyalomma* ticks, which dominate these zones, were especially involved in co‐infections. Similar patterns have been reported in northern Burkina Faso [55, 56], supporting the role of these dry savannah regions as hotspots for TBD transmission.

## 5. Conclusion

This study provides the most comprehensive molecular survey to date of tick‐borne microorganisms in Senegal, revealing a high infection rate (78.9%) and remarkable microbial diversity across ecological zones and host species. Using high‐throughput microfluidic real‐time PCR, we detected nine microorganisms, including both pathogens and endosymbionts in 12 tick species. The high prevalence of FLE (37.4%) across all zones and hosts suggests their widespread establishment and potential role in tick physiology. The detection of *A. ovis* and *A. bovis* for the first time in ticks in West Africa represents an important advance in understanding local pathogen ecology. While the predominance of *A. ovis* in *R. guilhoni* and *R. evertsi* highlights these species as potential vectors of small‐ruminant anaplasmosis.

The identification of *R. aeschlimannii*, *E. canis*, and *Coxiella* spp. in multiple tick species underscores the zoonotic and veterinary health risks associated with these vectors. The multivariable analysis further revealed that host type, rather than ecological zone or tick sex, significantly influenced infection status, emphasizing the central role of host–tick interactions in pathogen dynamics. Co‐infections were frequent (33.2%), particularly among *Hyalomma* species, confirming the complex microbial ecosystems sustained by these vectors.

Collectively, these findings highlight the extensive circulation of both pathogenic and symbiotic microorganisms in Senegalese tick populations and the need for integrated, molecularly based surveillance of TBPs in West Africa. Future studies should prioritize genome‐resolved metagenomic approaches to distinguish pathogenic *C. burnetii* from symbiotic *Coxiella*‐LE and to elucidate the functional roles of FLE in tick ecology. Such data are crucial for guiding vector control strategies, protecting livestock productivity, and mitigating zoonotic disease risks in a changing climate.

## Author Contributions

**Aliou Khoule:** investigation, validation, formal analysis, methodology, visualization, writing – original draft, writing – review and editing, data curation, software. **Clemence Galon**: formal analysis, methodology, visualization, writing – original draft, writing – review and editing, data curation, software. **Déthié Ngom:** investigation, methodology, data curation, software, validation, formal analysis, writing – review and editing. **Baye Bado Ndoye**: methodology, writing – review and editing. **Ousseynou Sene and Ibrahima Dia**: writing – review and editing. **Mawlouth Diallo:** methodology, validation. **Gamou fall**: funding acquisition. **Sara Moutailler and Diawo Diallo**: conceptualization, investigation, funding acquisition, writing – original draft, writing – review and editing, visualization, validation, methodology, formal analysis, project administration, resources, supervision, data curation.

## Funding

This research was funded by the National Institutes of Health (NIH), USA (Grant U01AI151758 and Subaward 3‐IPD‐NIH‐U01‐AS‐2022).

## Ethics Statement

Livestock owners were orally informed about the study objectives and procedures, and verbal consent was obtained before participation. Tick collections were carried out by trained veterinarians and zoologists, with owners assisting in animal restraint. After each session, animals received appropriate post‐collection care, including vitamins, antiparasitics, and antibiotics, if needed. Field staff strictly followed biosafety protocols by wearing protective clothing, using gloves, conducting regular tick checks, and handling ticks with fine‐tipped tweezers for safe storage in liquid nitrogen. All equipment was decontaminated after use. Livestock owners and local residents were also advised to avoid direct contact with animal fluids, especially during parturition, and to heat milk before consumption.

## Conflicts of Interest

The authors declare no conflicts of interest.

## Supporting Information

Additional supporting information can be found online in the Supporting Information section.

## Supporting information


**Supporting Information 1** Table S1. List of bacterial and protozoan pathogens targeted in this study using high‐throughput microfluidic real‐time PCR, including the corresponding genera, species, and total number of targets screened.

**Supporting Information 2** Table S2. Complete list of primers and probes used for high‐throughput microfluidic real‐time PCR assays, indicating the targeted organisms, genes, oligonucleotide sequences, amplicon sizes, and literature references.

**Supporting Information 3** Table S3. List of primers used for confirmation of selected pathogens by conventional and nested PCR, including target genes, primer sequences, expected product sizes, and references.

## Data Availability

Data are available upon request from the authors.
