# Supplementary material for: Detection of Multiple Microorganisms in Ruminant Ticks in Senegal Using High‐Throughput Microfluidic Real‐Time PCR
Source: Transbound Emerg Dis. 2026 Feb 20;2026:6292857. doi: 10.1155/tbed/6292857 (PMC12922542; doi:10.1155/tbed/6292857)
Supplement: Supplementary file 1 — Supporting Information 1 Table S1. List of bacterial and protozoan pathogens targeted in this study using high‐throughput microfluidic real‐time PCR, including the corresponding genera, species, and total number of targets screened. [file TBED-2026-6292857-s002.docx]

| **Table S1**. Bacteria and parasites targeted in our study | | |
| --- | --- | --- |
| Genus | Species | Numbers |
| *Borrelia* | *B. burgdorferi* senso stricto*, B. garinii, B. afzelii, B. valaisiana, B. lusitaniae, B. spielmanii, B. bissettii, B. miyamotoi.* | 8 |
| *Anaplasma* | *A. marginale, A. platys, A. phagocytophilum, A.ovis, A. centrale, A. bovis.* | 6 |
| *Ehrlichia* | *Ehrlichia canis, Neoehrlichia mikurensis* | 2 |
| *Rickettsia* | *R. conorii, R. slovaca, R. massiliae, R. felis, R. aeschlimannii, R. helvetica,* | 6 |
| *Bartonella* | *B. henselae* | 1 |
| *Francisella* | *F. tularensis, Francisella-*like endosymbionts | 2 |
| *Coxiella* | *C. burnettii, Coxiella-*like | 2 |
| *Babesia*  *Theileria*  *Hepatozoon* | *B. microti, B. ovis, B. bigemina, B. bovis, B. caballi, B. divergens, B. canis*  *Theileria* spp.  *Hepatozoon* spp. | 7  1  1 |
| 10 |  | 35 |
