# Supplementary material for: Detection of Multiple Microorganisms in Ruminant Ticks in Senegal Using High‐Throughput Microfluidic Real‐Time PCR
Source: Transbound Emerg Dis. 2026 Feb 20;2026:6292857. doi: 10.1155/tbed/6292857 (PMC12922542; doi:10.1155/tbed/6292857)
Supplement: Supplementary file 2 — Supporting Information 2 Table S2. Complete list of primers and probes used for high‐throughput microfluidic real‐time PCR assays, indicating the targeted organisms, genes, oligonucleotide sequences, amplicon sizes, and literature references. [file TBED-2026-6292857-s001.docx]

| **Table S2**. List of primers and probes used in this study for microfluidic real-time PCR | | | | | |
| --- | --- | --- | --- | --- | --- |
| **Organism** | **Targeted gene** | **Primers (F and R - 5'-3') and Probes (P)** | **Product length (bp)** | | **Reference** |
| *Borrelia burgdorferi* sensu stricto | *rpoB* | F-GCTTACTCACAAAAGGCGTCTT | | 83 | (Michelet et al., 2014) |
|  |  | R-GCACATCTCTTACTTCAAATCCT | |  |  |
|  |  | P-AATGCTCTTGGACCAGGAGGACTTTCA | |  |  |
| *Borrelia garinii* | *rpoB* | F-TGGCCGAACTTACCCACAAAA | | 88 | (Michelet et al., 2014) |
|  |  | R-ACATCTCTTACTTCAAATCCTGC | |  |  |
|  |  | P-TCTATCTCTTGAAAGTCCCCCTGGTCC | |  |  |
| *Borrelia afzelii* | *fla* | F-GGAGCAAATCAAGATGAAGCAAT | | 116 | (Michelet et al., 2014) |
|  |  | R-TGAGCACCCTCTTGAACAGG | |  |  |
|  |  | P-TGCAGCCTGAGCAGCTTGAGCTCC | |  |  |
| *Borrelia valaisiana* | *ospA* | F-ACTCACAAATGACAGATGCTGAA | | 135 | (Michelet et al., 2014) |
|  |  | R-GCTTGCTTAAAGTAACAGTACCT | |  |  |
|  |  | P-TCCGCCTACAAGATTTCCTGGAAGCTT | |  |  |
| *Borrelia lusitaniae* | *rpoB* | F-CGAACTTACTCATAAAAGGCGTC | | 87 | (Michelet et al., 2014) |
|  |  | R-TGGACGTCTCTTACTTCAAATCC | |  |  |
|  |  | P-TTAATGCTCTCGGGCCTGGGGGACT | |  |  |
| *Borrelia spielmanii* | *fla* | F-ATCTATTTTCTGGTGAGGGAGC | | 71 | (Michelet et al., 2014) |
|  |  | R-TCCTTCTTGTTGAGCACCTTC | |  |  |
|  |  | P-TTGAACAGGCGCAGTCTGAGCAGCTT | |  |  |
| *Borrelia bissetti* | *rpoB* | F-GCAACCAGTCAGCTTTCACAG | | 118 | (Michelet et al., 2014) |
|  |  | R-CAAATCCTGCCCTATCCCTTG | |  |  |
|  |  | P-AAAGTCCTCCCGGCCCAAGAGCATTAA | |  |  |
| *Borrelia myamotoi* | *glpQ* | F-CACGACCCAGAAATTGACACA | | 94 | (Michelet et al., 2014) |
|  |  | R-GTGTGAAGTCAGTGGCGTAAT | |  |  |
|  |  | P-TCGTCCGTTTTCTCTAGCTCGATTGGG | |  |  |
| *Borrelia* spp. | *23S rRNA* | F-GAGTCTTAAAAGGGCGATTTAGT | | 73 | (Michelet et al., 2014) |
|  |  | R-CTTCAGCCTGGCCATAAATAG | |  |  |
|  |  | P-AGATGTGGTAGACCCGAAGCCGAGT | |  |  |
| *Anaplasma marginale* | *msp1* | F-CAGGCTTCAAGCGTACAGTG | | 85 | (Michelet et al., 2014) |
|  |  | R-GATATCTGTGCCTGGCCTTC | |  |  |
|  |  | P-ATGAAAGCCTGGAGATGTTAGACCGAG | |  |  |
| *Anaplasma platys* | *groEL* | F-TTCTGCCGATCCTTGAAAACG | | 75 | (Michelet et al., 2014) |
|  |  | R-CTTCTCCTTCTACATCCTCAG | |  |  |
|  |  | P-TTGCTAGATCCGGCAGGCCTCTGC | |  |  |
| *Amnaplasma phagocytophilum* | *msp2* | F-GCTATGGAAGGCAGTGTTGG | | 77 | (Michelet et al., 2014) |
|  |  | R-GTCTTGAAGCGCTCGTAACC | |  |  |
|  |  | P-AATCTCAAGCTCAACCCTGGCACCAC | |  |  |
| *Anaplasma ovis* | *msp4* | F-TCATTCGACATGCGTGAGTCA | | 92 | (Michelet et al., 2014) |
|  |  | R-TTTGCTGGCGCACTCACATC | |  |  |
|  |  | P-AGCAGAGAGACCTCGTATGTTAGAGGC | |  |  |
| *Anaplasma centrale* | *groEL* | F-AGCTGCCCTGCTATACACG | | 79 | (Michelet et al., 2014) |
|  |  | R-GATGTTGATGCCCAATTGCTC | |  |  |
|  |  | P-CTTGCATCTCTAGACGAGGTAAAGGGG | |  |  |
| *Anaplasma bovis* | *groEL* | F-GGGAGATAGTACACATCCTTG | | 73 | (Sprong et al., 2019) |
|  |  | R-CTGATAGCTACAGTTAAGCCC | |  |  |
|  |  | P-AGGTGCTGTTGGATGTACTGCTGGACC | |  |  |
| *Anaplasma* spp. | *16S rRNA* | F-CTTAGGGTTGTAAAACTCTTTCAG | | 160 | (Gondard et al., 2020) |
|  |  | R-CTTTAACTTACCAAACCGCCTAC | |  |  |
|  |  | P-ATGCCCTTTACGCCCAATAATTCCGAACA | |  |  |
| *Ehrlichia canis* | *gltA* | F-GACCAAGCAGTTGATAAAGATGG | | 136 | (Gondard et al., 2020) |
|  |  | R-CACTATAAGACAATCCATGATTAGG | |  |  |
|  |  | P-ATTAAAACATCCTAAGATAGCAGTGGCTAAGG | |  |  |
| *Ehrlichia/Neoehrlichia* spp. | *16S rRNA* | F-GCAACGCGAAAAACCTTACCA | | 98 | (Gondard et al., 2020) |
|  |  | R-AGCCATGCAGCACCTGTGT | |  |  |
|  |  | P-AAGGTCCAGCCAAACTGACTCTTCCG | |  |  |
| Neoehrlichia mikurensis | *groEL* | F-AGAGACATCATTCGCATTTTGGA | | 96 | (Michelet et al., 2014) |
|  |  | R-TTCCGGTGTACCATAAGGCTT | |  |  |
|  |  | P-AGATGCTGTTGGATGTACTGCTGGACC | |  |  |
| *Rickettsia conorii* | *23S-5S ITS* | F-CTCACAAAGTTATCAGGTTAAATAG | | 118 | (Michelet et al., 2014) |
|  |  | R-CGATACTCAGCAAAATAATTCTCG | |  |  |
|  |  | P-CTGGATATCGTGGCAGGGCTACAGTAT | |  |  |
| *Rickettsia slovaca* | *23S-5S ITS* | F-GTATCTACTCACAAAGTTATCAGG | | 138 | (Michelet et al., 2014) |
|  |  | R-CTTAACTTTTACTACAATACTCAGC | |  |  |
|  |  | P-TAATTTTCGCTGGATATCGTGGCAGGG | |  |  |
| *Rickettsia massiliae* | *23S-5S ITS* | F-GTTATTGCATCACTAATGTTATACTG | | 128 | (Michelet et al., 2014) |
|  |  | R-GTTAATGTTGTTGCACGACTCAA | |  |  |
|  |  | P-TAGCCCCGCCACGATATCTAGCAAAAA | |  |  |
| *Rickettsia helvetica* | *23S-5S ITS* | F-AGAACCGTAGCGTACACTTAG | | 79 | (Michelet et al., 2014) |
|  |  | R-GAAAACCCTACTTCTAGGGGT | |  |  |
|  |  | P-TACGTGAGGATTTGAGTACCGGATCGA | |  |  |
| *Rickettsia aeshlimannii* | *23S-5S ITS* | F-CTCACAAAGTTATCAGGTTAAATAG | | 134 | (Sprong et al., 2019) |
|  |  | R-CTTAACTTTTACTACGATACTTAGCA | |  |  |
|  |  | P-TAATTTTTGCTGGATATCGTGGCGGGG | |  |  |
| *Rickettsia felis* | *orfB* | F-ACCCTTTTCGTAACGCTTTGC | | 163 | (Gondard et al., 2020) |
|  |  | R-TATACTTAATGCTGGGCTAAACC | |  |  |
|  |  | P-AGGGAAACCTGGACTCCATATTCAAAAGAG | |  |  |
| *Rickettsia* spp. | *gltA* | F-GTCGCAAATGTTCACGGTACTT | | 145 | (Michelet et al., 2014) |
|  |  | R-TCTTCGTGCATTTCTTTCCATTG | |  |  |
|  |  | P-TGCAATAGCAAGAACCGTAGGCTGGATG | |  |  |
| *Bartonella henselae* | *pap31* | F-CCGCTGATCGCATTATGCCT | | 107 | (Michelet et al., 2014) |
|  |  | R-AGCGATTTCTGCATCATCTGCT | |  |  |
|  |  | P-ATGTTGCTGGTGGTGTTTCCTATGCAC | |  |  |
| *Bartonella* spp. | *ssrA* | F-CGTTATCGGGCTAAATGAGTAG | | 118 | (Gondard et al., 2020) |
|  |  | R-ACCCCGCTTAAACCTGCGA | |  |  |
|  |  | P-TTGCAAATGACAACTATGCGGAAGCACGTC | |  |  |
| *Francisella tularensis* | *tul4* | F-ACCCACAAGGAAGTGTAAGATTA | | 76 | (Michelet et al., 2014) |
|  |  | R-GTAATTGGGAAGCTTGTATCATG | |  |  |
|  |  | P-AATGGCAGGCTCCAGAAGGTTCTAAGT | |  |  |
| *Francisella-*like endosymbiont | *fop4* | F-GGCAAATCTAGCAGGTCAAGC | | 91 | (Michelet et al., 2014) |
|  |  | R-CAACACTTGCTTGAACATTTCTAG | |  |  |
|  |  | P-AACAGGTGCTTGGGATGTGGGTGGTG | |  |  |
| *Coxiella burnetii* | *IS1111* | F-TGGAGGAGCGAACCATTGGT | | 86 | (Michelet et al., 2014) |
|  |  | R-CATACGGTTTGACGTGCTGC | |  |  |
|  |  | P-ATCGGACGTTTATGGGGATGGGTATCC | |  |  |
| *Coxiella-*like endosymbiont | *idc* | F-AGGCCCGTCCGTTATTTTACG | | 74 | (Michelet et al., 2014) |
|  |  | R-CGGAAAATCACCATATTCACCTT | |  |  |
|  |  | P-TTCAGGCGTTTTGACCGGGCTTGGC | |  |  |
| Apicomplexa | *18S* | F-TGAACGAGGAATGCCTAGTATG | | 104 | (Gondard et al., 2020) |
|  |  | R-CACCGGATCACTCGATCGG | |  |  |
|  |  | P-TAGGAGCGACGGGCGGTGTGTAC | |  |  |
| *Babesia microti* | *CCTeta* | F-ACAATGGATTTTCCCCAGCAAAA | | 145 | (Michelet et al., 2014) |
|  |  | R-GCGACATTTCGGCAACTTATATA | |  |  |
|  |  | P-TACTCTGGTGCAATGAGCGTATGGGTA | |  |  |
| *Babesia canis* (3 subspecies) | *18 rRNA* | F-TGGCCGTTCTTAGTTGGTGG | | 104 | (Michelet et al., 2014) |
|  |  | R-AGAAGCAACCGGAAACTCAAATA | |  |  |
|  |  | P-ACCGGCACTAGTTAGCAGGTTAAGGTC | |  |  |
| *Babesia ovis* | *18S rRNA* | F-TCTGTGATGCCCTTAGATGTC | | 92 | Michelet et al. 2014 |
|  |  | R-GCTGGTTACCCGCGCCTT | |  |  |
|  |  | P-TCGGAGCGGGGTCAACTCGATGCAT | |  |  |
| *Babesia bovis* | *CCTeta* | F-GCCAAGTAGTGGTAGACTGTA | | 100 | (Michelet et al., 2014) |
|  |  | R-GCTCCGTCATTGGTTATGGTA | |  |  |
|  |  | P-TAAAGACAACACTGGGTCCGCGTGG | |  |  |
| *Babesia caballi* | *Rap1* | F-GTTGTTCGGCTGGGGCATC | | 94 | (Michelet et al., 2014) |
|  |  | R-CAGGCGACTGACGCTGTGT | |  |  |
|  |  | P-TCTGTCCCGATGTCAAGGGGCAGGT | |  |  |
| *Babesia venatorum* (sp. EU1) | *18S rRNA* | F-GCGCGCTACACTGATGCATT | | 91 | (Michelet et al., 2014) |
|  |  | R-CAAAAATCAATCCCCGTCACG | |  |  |
|  |  | P-CATCGAGTTTAATCCTGTCCCGAAAGG | |  |  |
| *Babesia divergens* | *hsp70* | CTCATTGGTGACGCCGCTA | | 83 | (Michelet et al., 2014) |
|  |  | R-CTCCTCCCGATAAGCCTCTT | |  |  |
|  |  | P-AGAACCAGGAGGCCCGTAACCCAGA | |  |  |
| *Theileria* spp. | *18S rRNA* | F-GTCAGTTTTTACGACTCCTTCAG | | 213 | (Melis et al., 2024) |
|  |  | R-CCAAAGAATCAAGAAAGAGCTATC | |  |  |
|  |  | P-AATCTGTCAATCCTTCCTTTGTCTGGACC | |  |  |
| *Hepatozoon* spp. | *18S rRNA* | F-ATTGGCTTACCGTGGCAGTG | | 175 | (Gondard et al., 2020) |
|  |  | R-AAAGCATTTTAACTGCCTTGTATTG | |  |  |
|  |  | P-ACGGTTAACGGGGGATTAGGGTTCGAT | |  |  |
| *Ixodes ricinus* | *ITS2* | F-CGAAACTCGATGGAGACCTG | | 77 | (Michelet et al., 2014) |
|  |  | R-ATCTCCAACGCACCGACGT | |  |  |
|  |  | P-TTGTGGAAATCCCGTCGCACGTTGAAC | |  |  |
| *Dermacentor reticulatus* | *ITS2* | F-AACCCTTTTCCGCTCCGTG | | 83 | (Melis et al., 2024) |
|  |  | R-TTTTGCTAGAGCTCGACGTAC | |  |  |
|  |  | P-TACGAAGGCAAACAACGCAAACTGCGA | |  |  |
| *Dermacentor marginatus* | *ITS2* | F-GCACGTTGCGTTGTTTGCC | | 139 | (Michelet et al., 2014) |
|  |  | R-CCGCTCCGCGCAAGAATCT | |  |  |
|  |  | P-TTCGGAGTACGTCGAGCTCTAGCAGA | |  |  |
| Tick species | *16S rRNA* | F-AAATACTCTAGGGATAACAGCGT | | 99 | (Gondard et al., 2020) |
|  |  | R-TCTTCATCAAACAAGTATCCTAATC | |  |  |
|  |  | P-CAACATCGAGGTCGCAAACCATTTTGTCTA | |  |  |
| *Escherichia coli* | *eae* | F-CATTGATCAGGATTTTTCTGGTGATA | | 102 | (Michelet et al., 2014) |
|  |  | R-CTCATGCGGAAATAGCCGTTA | |  |  |
|  |  | P-ATAGTCTCGCCAGTATTCGCCACCAATACC | |  |  |
