# Supplementary material for: Detection of Multiple Microorganisms in Ruminant Ticks in Senegal Using High‐Throughput Microfluidic Real‐Time PCR
Source: Transbound Emerg Dis. 2026 Feb 20;2026:6292857. doi: 10.1155/tbed/6292857 (PMC12922542; doi:10.1155/tbed/6292857)
Supplement: Supplementary file 3 — Supporting Information 3 Table S3. List of primers used for confirmation of selected pathogens by conventional and nested PCR, including target genes, primer sequences, expected product sizes, and references. [file TBED-2026-6292857-s003.docx]

| **Table S3.** List of primers used in this study for confirmation using nested and conventional PCR | | | | | |
| --- | --- | --- | --- | --- | --- |
| **Pathogen** | **Targeted gene** | **Primer name** | **Primer (5'-3')** | **Product length (bp)** | **References** |
| *Borrelia* spp. | *flaB* | FlaB280F | GCAGTTCARTCAGGTAACGG | 645 | (Loh et al., 2016) |
|  |  | FlaRL | GCAATCATAGCCATTGCAGATTGT |  |  |
|  |  | flaB_737F | GCATCAACTGTRGTTGTAACATTAACAGG | 407 |  |
|  |  | FlaLL | ACATATTCAGATGCAGACAGAGGT |  |  |
| *Rickettsia* spp. | *gltA* | Rsfg877 | GGGGGCCTGCTCACGGCGG | 381 | (Regnery R L et al., 1991) |
|  |  | Rsfg1258 | ATTGCAAAAAGTACAGTGAACA |  |  |
|  | *ompB* | Rc.rompB.4362p | GTCAGCGTTACTTCTTCGATGC | 475 | (Choi et al., 2023) |
|  |  | Rc.rompB.4836n | CCGTACTCCATCTTAGCATCAG |  |  |
|  |  | Rc.rompB.4,496p | CCAATGGCAGGACTTAGCTACT | 267 |  |
|  |  | Rc.rompB.4,762n | AGGCTGGCTGATACACGGAGTAA |  |  |
| *Hepatozoon* spp. | *18S rRNA* | HepF | ATA CAT GAG CAA AAT CTC AAC | 660 | (Inokuma et al., 2002) |
|  |  | HepR | CTTATTATTCCATGCTGCAG |  |  |
|  |  | HepNF | GGTATGGTATTGGCTTACCG | 309 | (de Azevedo Gomes et al., 2018) |
|  |  | HepNR | CGAGCTTTTTAACTGCAACA |  |  |
| *Anaplasma* and *Ehrlichia* spp. | *18S rRNA* | EHR1 | GAACGAACGCTGGCGGCAAGC  AGTA(T/C)CG(A/G)ACCAGATAGCCGC  TGCATAGGAATCTACCTAGTAG | 693 | (Rar et al., 2005) |
|  |  | EHR2 |  |  |  |
|  |  | EHR3 |  | 592 |  |
| *Babesia* spp. | *18S rRNA* | BTH18S | GGG CTA ATA CAW GTT CGA G | 1650 | (Jouglin et al., 2017) |
|  |  | CryptoRtick | GAA TGA TCC TTC TGC AGG TTC ACC TAC |  |  |
|  |  | BABGF2 | GYY TTG TAA TTG GAA TGA TGG | 550 |  |
|  |  | BABGR2 | CCA AAG ACT TTG ATT TCT CTC |  |  |
| Babesia/  Theileria /Hepatozoon spp | 18S rRNA | BTH 18S 1st F  BTH 18S 1st R  BTH 18S 2nd F  BTH 18S 2nd R | GTGAAACTGCGAATGGCTCATTAC  AAGTGATAAGGTTCACAAAACTTCCC  GGCTCATTACAACAGTTATAGTTTATTTG  CGGTCCGAATAATTCACCGGAT | 1500 | (Masatani et al., 2017) |
| Coxiella | *16SrDNA* | Cox 16SF1  Cox 16SR1  Cox 16SF2  Cox 16SR2 | CGTAGGAATCTACCTTRTAGWGG  ACTYYCCAACAGCTAGTTCTCA  TGAGAACTAGCTGTTGGRRAGT  GCCTACCCGCTTCTGGTACAATT | 719  624 | (Duron et al., 2017) |
| Bartonella | *gltA* | bart781  bart1137 | GGG GAC CAG CTC ATG GTG G-3  AAT GCA AAA AGA ACA GTA AAC A | 380 | (Norman et al., 1995) |
| Neoehrlichia | *groEL* | NM 1152as  NM 128s | TTC TAC TTT GAA CAT TTG AAG AAT TAC TAT  AAC AGG TGA AAC ACT AGA TAA GTC CAT | 1024 | (Diniz et al., 2011) |
|  |  |  |  |  |  |
